# Comparative long-term prognosis of acute myocardial infarction and acute aortic dissection in a population-based registry

**DOI:** 10.1093/ehjopen/oeag094

**Published:** 2026-05-30

**Authors:** Yuichi Sawayama, Akiko Harada, Naoyuki Takashima, Takashi Yamamoto, Yosuke Higo, Kensuke Takabayashi, Wataru Shioyama, Takako Fujii, Sachiko Tanaka-Mizuno, Yoshikuni Kita, Katsuyuki Miura, Kazumichi Yoshida, Kazuhiko Nozaki, Tomoaki Suzuki, Yoshihisa Nakagawa

**Affiliations:** Department of Cardiovascular Medicine, Shiga University of Medical Science, Shiga 520-2192, Japan; NCD Epidemiology Research Center, Shiga University of Medical Science, Shiga 520-2192, Japan; NCD Epidemiology Research Center, Shiga University of Medical Science, Shiga 520-2192, Japan; Department of Epidemiology for Community Health and Medicine, Kyoto Prefectural University of Medicine, Kyoto 602-8566, Japan; Department of Cardiovascular Medicine, Shiga University of Medical Science, Shiga 520-2192, Japan; Department of Cardiovascular Medicine, Kohka Public Hospital, Shiga 528-0074, Japan; Department of Cardiovascular Medicine, Shiga University of Medical Science, Shiga 520-2192, Japan; Department of Cardiovascular Medicine, Shiga University of Medical Science, Shiga 520-2192, Japan; Department of Cardiovascular Medicine, Shiga University of Medical Science, Shiga 520-2192, Japan; NCD Epidemiology Research Center, Shiga University of Medical Science, Shiga 520-2192, Japan; Department of Preventive Medicine and Public Health, Fukuoka University, Fukuoka 814-0180, Japan; NCD Epidemiology Research Center, Shiga University of Medical Science, Shiga 520-2192, Japan; Laboratory of Epidemiology and Prevention, Kobe Pharmaceutical University, Hyogo 658-8558, Japan; NCD Epidemiology Research Center, Shiga University of Medical Science, Shiga 520-2192, Japan; Faculty of Nursing Science, Tsuruga Nursing University, Fukui 914-0814, Japan; NCD Epidemiology Research Center, Shiga University of Medical Science, Shiga 520-2192, Japan; Department of Public Health, Shiga University of Medical Science, Shiga 520-2192, Japan; Department of Neurosurgery, Shiga University of Medical Science, Shiga 520-2192, Japan; Department of Neurosurgery, Shiga University of Medical Science, Shiga 520-2192, Japan; Division of Cardiovascular Surgery, Shiga University of Medical Science, Shiga 520-2192, Japan; Department of Cardiovascular Medicine, Shiga University of Medical Science, Shiga 520-2192, Japan

**Keywords:** Acute aortic dissection, Acute myocardial infarction, Mortality

## Abstract

**Aims:**

Acute myocardial infarction (AMI) and acute aortic dissection (AAD) are major cardiovascular emergencies with high mortality; however, their long-term prognoses have rarely been directly compared within the same population. Understanding differences in real-world outcomes between these conditions is important for patient informing and evaluation of cardiovascular care.

**Methods and results:**

We analysed 1923 patients enrolled in the Shiga Stroke and Heart Attack Registry, a population-based registry covering an entire Shiga Prefecture in Japan, who developed AMI or AAD between 2014 and 2015. The primary outcome was all-cause death, and the secondary outcome was cardiovascular death during 5-year follow-up. Hazard ratios (HRs) were estimated using multivariable Cox models adjusted for age and sex. Landmark analyses beyond 30 days evaluated outcomes among early survivors. Of the total cohort, 1550 were AMI [ST-elevation myocardial infarction (MI): *n* = 902 (58%); non-ST-elevation MI: *n* = 464 (30%); and sudden cardiac death due to MI: *n* = 184 (12%)], and 373 were AAD [type A: *n* = 219 (59%); type B: *n* = 154 (41%)]. Five-year all-cause mortality was higher in AAD than AMI [50.0% vs. 35.4%; adjusted HR 1.53, 95% confidence interval (CI) 1.29–1.83; *P* < 0.001]. Cardiovascular mortality was also higher in AAD (38.7% vs. 27.4%; adjusted HR 1.41, 95% CI 1.17–1.71; *P* < 0.001). Landmark analysis showed that excess mortality in AAD was mainly driven by early deaths, whereas cardiovascular mortality among 30-day survivors was similar between diseases.

**Conclusion:**

In this population-based study in Japan, AAD was associated with worse 5-year survival than AMI. Among acute-phase survivors, long-term cardiovascular outcomes were comparable.

## Graphical Abstract

**Graphical Abstract:**
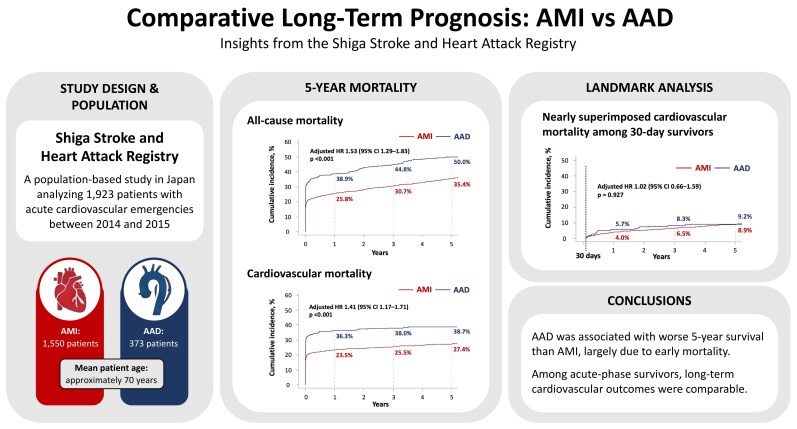

## Introduction

Acute myocardial infarction (AMI) remains one of the leading causes of death worldwide, despite substantial advances in cardiovascular therapeutics.^1^ Both modifiable risk factors (e.g. smoking, hypertension, diabetes, dyslipidaemia, and obesity) and non-modifiable factors (e.g. age, gender, and family history) contribute to the development of the disease.^2^ These factors are also recognized as general risk factors for atherosclerotic disease and may partly contribute to the occurrence of acute aortic dissection (AAD), a catastrophic cardiovascular emergency associated with high mortality.^3^

Although AMI and AAD share several clinical risk factors and may present with similar initial clinical features, particularly subjective symptoms at onset, their underlying pathophysiological mechanisms differ substantially. Acute myocardial infarction is primarily caused by atherosclerotic plaque rupture or erosion in the coronary arteries,^4^ whereas AAD results from a tear in the aortic wall leading to separation of the aortic layers.^5^ Importantly, AMI represents a cardiovascular condition for which management has been highly standardized, including time-sensitive acute reperfusion strategies (e.g. door-to-balloon time) and well-established long-term secondary prevention such as lipid control. In contrast, although acute-phase treatment strategies for AAD are relatively well defined, critical aspects such as optimization of time-to-treatment and chronic-phase management remain less standardized and continue to evolve.

These differences in the maturity of treatment systems, in addition to distinct pathophysiological mechanisms, may lead to disparities in both acute-phase mortality and long-term prognosis. To date, several studies have investigated the long-term prognosis of AMI^6–10^ and AAD^11–14^ separately. However, no available data exist that directly compared the long-term mortality of AMI and AAD within a single population-based cohort. Given that both AMI and AAD present as acute chest pain syndromes in similar clinical settings, a comparative analysis within a unified, community-based population may provide a comprehensive overview of acute cardiovascular emergencies. Such an approach may help identify gaps in current AAD management by benchmarking it against the more established care pathways of AMI and ultimately support improved risk stratification, clinical decision-making, and patient communication.

The Shiga Stroke and Heart Attack Registry (SSHR) is an ongoing, multicentre, population-based registry in Shiga Prefecture, Japan, which systematically tracks both the acute phase and long-term outcomes of patients with AMI and AAD. Using this comprehensive population-based registry, we aimed to directly compare the long-term prognosis of AMI and AAD within the same community, providing real-world insights into the temporal patterns of mortality following major cardiovascular emergencies.

## Methods

### Data collection

**Figure 1:**
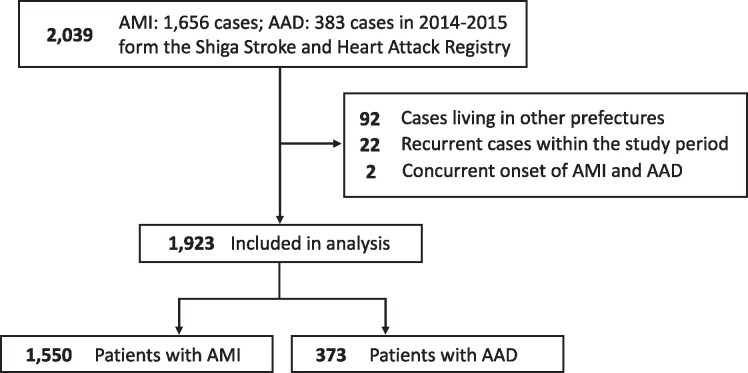
Flowchart of study enrolment. AAD, acute aortic dissection; AMI, acute myocardial infarction.

The study design and recruitment details have been previously reported.^15,16^ In brief, the registry was designed to evaluate the incidence and prognosis of acute cerebro-cardiovascular diseases, including stroke, MI, and aortic disease, in Shiga Prefecture, which has a population of approximately 1.4 million. A total of 2039 cases of AMI or AAD with onset between January 2014 and December 2015 were followed until December 2019. In this analysis, we excluded cases living in other prefectures (*n* = 92), recurrent cases within the study period (*n* = 22), and concurrent onset of AMI and AAD (*n* = 2). Finally, 1923 cases were included in the present analysis (*Figure 1*). The SSHR has been approved by the Institutional Review Board of Shiga University of Medical Science (R2011-86).

### Definition of disease types

We defined AMI according to the fourth universal definition of myocardial infarction (UDMI) and classified it into the following MI types: Type 1 (MI precipitated by atherosclerotic plaque disruption), Type 2 (MI because of mismatch between oxygen supply and demand), and Type 3 (cardiac death suggestive of MI, with death before blood samples for biomarkers could be obtained or before increases in cardiac biomarkers could be identified).^17^ When the troponin level was not available, the WHO-MONICA criteria were applied (i.e. creatine–phosphokinase and/or creatine–phosphokinase–MB fraction at least twice the normal limit). We classified ST-elevation myocardial infarction (STEMI) or non-STEMI (NSTEMI) according to the electrocardiogram findings. Acute aortic dissection was defined as any imaging examination within 2 weeks of onset and classified into two subtypes according to the Stanford classification system; type A AAD involves the ascending aorta, regardless of the site of the primary intimal tear, whereas other cases are diagnosed as type B AAD.^18^

### Follow-up of outcomes

The primary outcome was death due to any cause. The secondary outcome was cardiovascular death. Cardiovascular death was defined according to the 2018 Academic Research Consortium 2 criteria.^19^ We used a death certificate from 2014 to 2019 of Shiga Prefecture to help identify deaths with the approval of the Japanese Ministry of Health, Labour and Welfare. Information on participants’ place of residence and cause of death was collected. Causes of death were classified based on the descriptions recorded on the certificates and the International Classification of Diseases, 10th edition (ICD-10). The codes used for cardiovascular death were as follows: coronary artery disease (I20–I25); valvular heart disease (I34–I39); cardiomyopathy (I42); heart failure (I50); stroke (I60–I69); and aortic disease (I70–I74).

### Statistical analysis

Summary statistics for demographic and clinical characteristics are provided for the overall cohort and for subgroups with AMI and AAD. Categorical data were reported as numbers and percentages, and they were compared using the chi-square or Fisher’s exact test. Continuous data were expressed as the mean and standard deviation or the median and interquartile range (IQR), depending on the data distribution. Student’s *t*-test or the Mann–Whitney U test was used for the comparison.

Cumulative incidence of death at 5 years for patients with AMI and AAD were estimated using Kaplan–Meier methods. Log-rank tests were used to compare survival rates across diseases. Hazard ratios (HRs) and 95% CIs were calculated using a multivariable Cox proportional hazards model, adjusting for age and sex. We then divided them into those who received interventional treatment and those who did not and compared the mortality. Additionally, given that both AMI and AAD are associated with substantial early mortality, evaluating long-term outcomes requires careful consideration of early survival to accurately characterize disease-specific trajectories. Therefore, to minimize differences in early-phase mortality among diseases, a landmark analysis was performed beyond 30 days. Moreover, logistic regression models were used to estimate age-specific mortality rates within 30 days and landmark mortality from 30 days to 5 years for each disease. All reported *P*-values were two-sided, and a value of <0.05 was considered statistically significant. We analysed all data using SAS software, version 9.4 (SAS Institute, Inc., Cary, North Carolina, USA).

## Results

### Patient characteristics

We analysed data from 1923 patients in the SSHR. Of these, 1550 were AMI patients [STEMI: *n* = 902 (58%); NSTEMI: *n* = 464 (30%); sudden cardiac death because of MI: *n* = 184 (12%)], and 373 were AAD patients [type A: *n* = 219 (59%); type B: *n* = 154 (41%)]. The patients’ baseline clinical characteristics are presented in *Table 1*. Patients with AMI and AAD were both approximately 70 years old (AMI 71 years vs. AAD 73 years), and men were more prevalent in AMI patients than in AAD patients (AMI 70% vs. AAD 52%). Conventional atherosclerotic factors, including current smoking, diabetes, and dyslipidaemia, showed a higher rate in AMI patients than AAD patients. The prevalence of hypertension was similar in the two groups, observed in approximately 60%. Systolic blood pressure on hospital arrival was higher in AAD patients (148 ± 40 mmHg) than AMI patients (140 ± 34 mmHg), while diastolic blood pressure and pulse rate were comparable between the two groups. The detailed patient characteristics by each subgroup are described in Supplementary material online, *Table S1*. Patients with type A AAD were characterized by an older age, a higher proportion of women, and higher rates of cardiopulmonary arrest.

**Table 1:** Baseline characteristics of patients with AMI and AAD

	Overall (*n* = 1923)	AMI (*n* = 1550)	AAD (*n* = 373)	*P*-value
Age, years	71.6 ± 13.4	71.3 ± 13.2	72.9 ± 13.9	0.034
Men	1283 (66.7%)	1090 (70.3%)	193 (51.7%)	<0.001
Height	161.0 ± 10.2 (*n* = 1600)	161.4 ± 10.0 (*n* = 1317)	159.2 ± 10.7 (*n* = 283)	0.001
BMI	23.5 ± 4.0 (*n* = 1570)	23.6 ± 3.8 (*n* = 1296)	23.3 ± 4.7 (*n* = 274)	0.208
Current smoking	533/1620 (32.9%)	454/1336 (34.0%)	79/284 (27.8%)	0.045
Current drinking	585/1530 (38.2%)	496/1269 (39.1%)	89/261 (34.1%)	0.131
Living alone	215 (11.2%)	172 (11.1%)	43 (11.5%)	0.812
Comorbidities				
Hypertension	1142/1840 (62.1%)	914/1486 (61.5%)	228/354 (64.4%)	0.312
Diabetes	686/1845 (37.2%)	613/1493 (41.1%)	73/352 (20.7%)	<0.001
Dyslipidaemia	1074/1847 (58.2%)	939/1493 (62.9%)	135/354 (38.1%)	<0.001
Prior MI	138/1835 (7.5%)	130/1484 (8.8%)	8/351 (2.3%)	<0.001
Prior AoD	73/1828 (4.0%)	32/1481 (2.2%)	41/347 (11.8%)	<0.001
Prior ischaemic stroke	167/1838 (9.1%)	139/1486 (9.4%)	28/352 (8.0%)	0.411
Prior intracerebral bleeding	39/1833 (2.1%)	26/1482 (1.8%)	13/351 (3.7%)	0.023
Prior PCI	220/1829 (12.0%)	206/1478 (13.9%)	14/351 (4.0%)	<0.001
Prior CABG	17/1824 (0.9%)	17/1473 (1.2%)	0/351 (0%)	0.043
Blood pressure and heart rate on hospital arrival				
SBP, mmHg	141.3 ± 34.8 (*n* = 1603)	139.8 ± 33.5 (*n* = 1319)	148.1 ± 39.5 (*n* = 284)	<0.001
DBP, mmHg	82.2 ± 22.3 (*n* = 1596)	82.2 ± 21.7 (*n* = 1312)	82.0 ± 24.9 (*n* = 284)	0.894
Pulse rate/min	79.4 ± 21.6 (*n* = 1603)	79.5 ± 22.0 (*n* = 1322)	79.0 ± 19.5 (*n* = 281)	0.699
States at hospital arrival				
CPA	353/1892 (18.7%)	250/1519 (16.5%)	103/373 (27.6%)	<0.001
Japan coma scale				0.002
I	1454/1818 (80.0%)	1204/1477 (81.5%)	250/341 (73.3%)	
II	87/1818 (4.8%)	63/1477 (4.3%)	24/341 (7.0%)	
III	277/1818 (15.2%)	210/1477 (14.2%)	67/341 (19.7%)	

Data are presented as mean ± standard deviation for continuous variables or count (%) for categorical variables. The *P*-values were calculated between patients with AMI and those with AAD. For categorical variables, the chi-square test was applied, and continuous variables were compared using Student’s *t*-test.

AoD, aortic diseases; AAD, acute aortic dissection; AMI, acute myocardial infarction; CABG, coronary artery bypass graft; CPA, cardiopulmonary arrest; DBP, diastolic blood pressure; eGFR, estimated glomerular filtration rate; ER, emergency room; MI, myocardial infarction. PCI, percutaneous coronary intervention; SBP, systolic blood pressure.

### Long-term mortality

**Figure 2:**
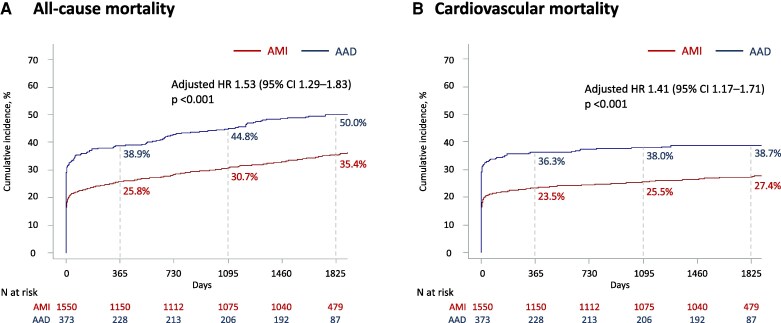
Cumulative incidence of all-cause and cardiovascular mortality in patients with AMI and AAD. Kaplan–Meier curves between patients with AMI (red) and AAD (blue). Cumulative incidence of death from (*A*) any cause and (*B*) cardiovascular diseases. Dashed vertical lines indicate the cumulative event rates at 1-, 3-, and 5-year time points. Hazard ration (HR) and 95% confidence interval (CI) were estimated using a multivariable Cox proportional hazards model adjusting for age and sex, with AMI as the reference. AAD, acute aortic dissection; AMI, acute myocardial infarction.

Among all analysed patients, the cumulative incidence of death from any cause at 1, 3, and 5 years were 25.8%, 30.7%, and 35.4% for AMI and 38.9%, 44.8%, and 50.0% for AAD, respectively (*Figure 2A*). After adjustment for age and sex, AAD patients showed a 1.5-fold higher risk of all-cause mortality compared with AMI patients (adjusted HR 1.53, 95% CI 1.29–1.83, *P* < 0.001). For cardiovascular death, the cumulative rates at 1, 3, and 5 years were 23.5%, 25.5%, and 27.4% for AMI and 36.3%, 38.0%, and 38.7% for AAD, with a significantly higher rate in AAD patients (adjusted HR 1.41, 95% CI 1.17–1.71, *P* < 0.001; *Figure 2B*).

Among the subtypes in the overall cohort (including both treated and untreated patients), patients with type A AAD presented the highest 5-year cumulative incidence of death from any cause at 60.5%, followed by type B AAD at 35.1%, STEMI at 28.8%, and NSTEMI at 22.6% (see Supplementary material online, *Figure S1A*). As for cardiovascular death, the cumulative 5-year rate was also highest in type A AAD at 52.7%, followed by STEMI at 20.0%, type B AAD at 18.8%, and NSTEMI at 12.7% (see Supplementary material online, *Figure S1B*).

### Landmark analysis beyond 30 days

**Figure 3:**
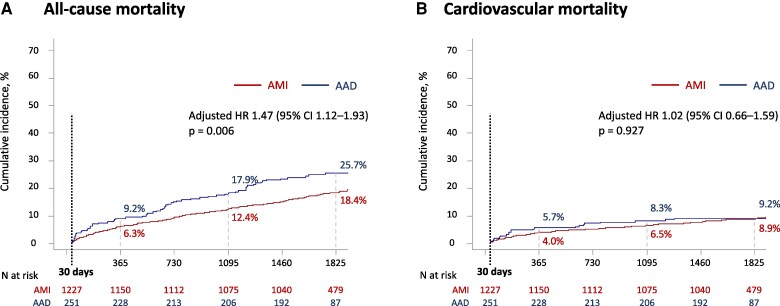
Landmark analysis beyond 30 days. Patients who died within 30 days after the onset were excluded from the landmark analysis beyond 30 days. Cumulative incidence of death from (*A*) any cause and (*B*) cardiovascular diseases. Hazard ratios (HRs) and 95% confidence intervals (CIs) were calculated by the multivariable Cox proportional hazard regression model adjusting for age and sex, with AMI as the reference. The colour coding is as follows: AMI (red) and AAD (blue). AAD, acute aortic dissection; AMI, acute myocardial infarction.

In the landmark analysis beyond 30 days, the cumulative incidence of death from any cause at 1, 3, and 5 years were also significantly higher in AAD patients than AMI patients (adjusted HR 1.47, 95% CI 1.12–1.93, *P* = 0.006; *Figure 3A*). In contrast, for cardiovascular death, the Kaplan–Meier curves were nearly identical between AAD and AMI patients (adjusted HR 1.02, 95% CI 0.66–1.59, *P* = 0.927; *Figure 3B*). By subtype, the cumulative incidence of death from any cause shows that patients with type B AAD have higher mortality than those with type A AAD, followed by nearly overlapping curves in patients with STEMI and NSTEMI (see Supplementary material online, *Figure S2A*). For cardiovascular death, patients with type B AAD exhibited a higher mortality rate, whereas the curves for the other subtypes were nearly superimposed (see Supplementary material online, *Figure S2B*).

### Impact of interventional treatment on long-term mortality

**Figure 4:**
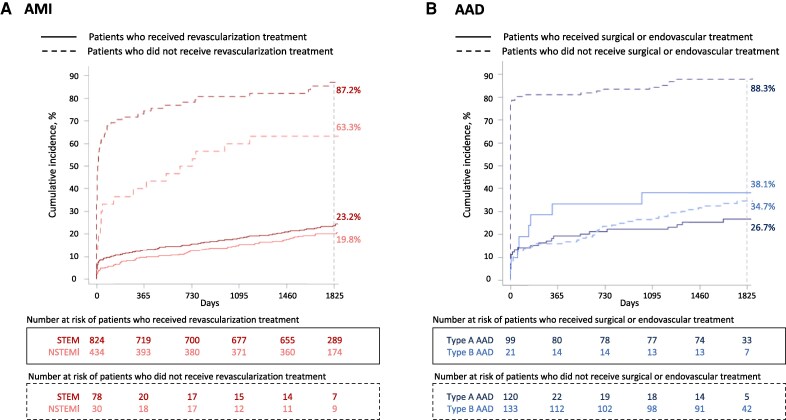
Cumulative incidence of all-cause mortality by treatment strategy in patients with AMI and AAD. Cumulative incidence curves for all-cause mortality according to treatment strategy in patients with (*A*) AMI and (*B*) AAD. Solid lines indicate treated patients (revascularization for AMI; surgical or endovascular treatment for AAD), and dashed lines indicate untreated patients. The colour coding is as follows: STEMI (dark red), NSTEMI (light red), type A AAD (dark blue), and type B AAD (light blue). AAD, acute aortic dissection; AMI, acute myocardial infarction; NSTEMI, non-ST-elevation myocardial infarction; STEMI, ST-elevation myocardial infarction.

*Table 2* summarizes the all-cause and cardiovascular mortality according to disease subtypes and intervention status. Among patients with AMI, those who did not undergo revascularization had markedly poorer outcomes, with 5-year all-cause mortality at 87.2% for STEMI and 63.3% for NSTEMI, compared with 23.2% and 19.8%, respectively, among those who underwent the procedure [adjusted HRs: 3.55 (95% CI, 2.56–4.91) and 3.38 (95% CI, 1.96–5.82); *Figure 4A*, Supplementary material online, *Table S2*]. Similarly, in type A AAD, nonsurgically treated patients had a 5-year all-cause mortality at 88.3% vs. 26.7% among those receiving surgical treatment (adjusted HR 6.24, 95% CI, 3.90–10.0; *Figure 4B*, Supplementary material online, *Table S2*). In contrast, in type B AAD, patients undergoing surgical or endovascular intervention showed slightly higher 5-year all-cause mortality (38.1%) than those managed conservatively (34.7%; adjusted HR 0.65, 95% CI, 0.31–1.39; *Figure 4B*, Supplementary material online, *Table S2*). A similar trend was observed for cardiovascular mortality.

**Table 2:** All-cause and cardiovascular mortality according to disease subtypes and intervention Status

	KM rate of all-cause death			KM rate of cardiovascular death		
	At 1 year	At 3 years	At 5 years	At 1 year	At 3 years	At 5 years
Type A AAD (*n* = 219)	117 (53.4)	124 (56.6)	132 (60.5)	112 (51.2)	114 (52.2)	115 (52.7)
With interventions (*n* = 99)	19 (19.2)	22 (22.2)	26 (26.7)	15 (15.2)	16 (16.3)	17 (17.4)
Without interventions (*n* = 120)	98 (81.7)	102 (85.0)	106 (88.3)	97 (80.9)	98 (81.8)	98 (81.8)
Type B AAD (*n* = 154)	28 (18.2)	43 (27.9)	53 (35.1)	23 (15.1)	28 (18.0)	29 (18.8)
With interventions (*n* = 21)	7 (33.3)	8 (38.1)	8 (38.1)	5 (25.5)	6 (30.8)	6 (30.8)
Without interventions (*n* = 133)	21 (15.8)	35 (26.3)	45 (34.7)	18 (13.6)	22 (16.9)	23 (17.8)
STEMI (*n* = 902)	163 (18.1)	210 (23.3)	253 (28.8)	139 (15.5)	159 (17.8)	176 (20.0)
With interventions (*n* = 824)	105 (12.7)	147 (17.8)	186 (23.2)	86 (10.5)	103 (12.6)	118 (14.8)
Without interventions (*n* = 78)	58 (74.4)	63 (80.8)	67 (87.2)	53 (69.7)	56 (74.6)	58 (78.5)
NSTEMI (*n* = 464)	53 (11.4)	81 (17.5)	103 (22.6)	40 (8.7)	49 (10.8)	57 (12.7)
With interventions (*n* = 434)	41 (9.5)	63 (14.5)	84 (19.8)	30 (7.0)	36 (8.4)	44 (10.5)
Without interventions (*n* = 30)	12 (40.0)	18 (60.0)	19 (63.3)	10 (34.2)	13 (46.9)	13 (46.9)

Values are *n* (%) according to Kaplan–Meier (KM) estimate.

AAD, acute aortic dissection; NSTEMI, non-ST-elevation myocardial infarction; STEMI, ST-elevation myocardial infarction.

### Age impact on mortality

To assess the potential impact of age on mortality, we estimated age-specific mortality separately for patients with AMI and AAD. In the acute phase within 30 days after onset, mortality rates increased exponentially with age in both groups, with a particularly pronounced trend in patients with AAD. At any given age, patients with AAD consistently exhibited higher mortality than those with AMI (see Supplementary material online, *Figure S3A*). Among patients who survived the initial 30 days, both groups showed an age-related increase in mortality over a follow-up of up to 5 years. However, the mortality rates were nearly equivalent between AAD and AMI patients across all age groups (Supplementary material online, *Figure S3B*).

## Discussion

This population-based study directly compared long-term outcomes between patients with AAD and AMI within the same regional healthcare system. The main findings are threefold. First, patients with AAD had substantially higher long-term all-cause and cardiovascular mortality than those with AMI. Second, the excess mortality associated with AAD was largely driven by early deaths occurring within the acute phase. Third, among patients who survived beyond 30 days, cardiovascular mortality became comparable between the two diseases. To our knowledge, this is the first study to provide a direct long-term prognostic comparison between AAD and AMI using a population-based registry covering an entire prefecture, offering real-world insight beyond the acute phase.

To date, few population-based studies have reported long-term outcomes after AMI. In contrast, several hospital-based registries, such as CREDO-Kyoto AMI (Coronary Revascularization Demonstrating Outcome study in Kyoto Acute Myocardial Infarction) Registry^6^ in Japan, OACIS (Osaka Acute Coronary Insufficiency) Study^7^ in Japan, and CRUSADE (Can rapid risk stratification of unstable angina patients suppress adverse outcomes with early implementation of the ACC/AHA guidelines) study^8^ in the USA, have reported 5-year mortality rates ranging from 10% to 50%. Given that these studies are based on highly selected hospital cohorts, they do not fully represent case fatality in the regional population. In this context, our population-based registry demonstrated a 5-year all-cause mortality at 35.4% in patients with AMI (28.8% for STEMI and 22.6% for NSTEMI), reflecting real-world outcomes in a community-dwelling population in Japan. Notably, in our cohort, the mean age was similar (approximately 70 years) between STEMI and NSTEMI patients. This contrasts with the commonly reported older age in NSTEMI populations and may reflect the advanced ageing of this community-based registry in Japan. In particular, the ageing of the STEMI population, along with the inclusion of all consecutive patients regardless of treatment strategy (including very elderly patients who did not undergo intervention), likely contributed to this attenuation of age differences.

For AAD, several population-based registries have reported long-term outcomes. In Iceland, a nationwide study including the entire population (approximately 300 000) during 2007–13 reported a 5-year all-cause mortality at 48.4%.^11^ In Oxfordshire, UK (population approximately 90 000; 2002–12), comprehensive population-based ascertainment yielded a 5-year all-cause mortality at 64.5%.^12^ Our population-based registry documented a 5-year all-cause mortality at 50.0% (type A 60.5%, type B 35.1%) in 2014–15, a period reflecting modern diagnostic and therapeutic practice, consistent with these previous studies in demonstrating high long-term mortality.

In our comparative analysis, patients with AAD had significantly higher all-cause and cardiovascular mortality than those with AMI, with 1.5-fold and 1.4-fold increased HRs, respectively, even after adjusting for age and sex. This excess mortality associated with AAD was mainly driven by type A AAD, particularly during the acute phase, when approximately half of the patients died within 30 days of onset. Moreover, our data showed that patients with AAD consistently exhibited higher mortality than those with AMI across all age groups. Importantly, even among younger individuals, the inherent seriousness of AAD remained more profound than that of AMI. While AMI outcomes have been substantially improved through highly standardized, time-sensitive treatment systems (e.g. door-to-balloon protocols), the management of AAD remains less uniformly structured. In our cohort, early mortality in AAD remained markedly high, highlighting persistent challenges in timely diagnosis and access to emergent surgical care. These findings underscore the need for more streamlined and time-sensitive care pathways for AAD, analogous to those established for AMI.

Approximately 8% of patients with AMI did not undergo revascularization because of treatment refusal or clinical ineligibility, and these non-interventionally managed patients experienced markedly high mortality (5-year mortality: 87% for STEMI and 63% for NSTEMI). Similarly, among patients with type A AAD, those who did not receive surgical treatment (55% of all type A AAD cases) had extremely poor outcomes, with mortality reaching 90% during the acute phase. In contrast, surgically treated type A AAD patients demonstrated favourable long-term outcomes. Accordingly, in the landmark analysis beyond 30 days, the Kaplan–Meier curves for all-cause mortality in patients with type A AAD fell below those with type B AAD, consistent with data from the International Registry of Acute Aortic Dissection (IRAD).^13,14^ In our cohort, patients with type B AAD had a higher prevalence of cardiovascular risk factors, such as smoking, hypertension, and dyslipidaemia, which may have contributed to their less favourable long-term cardiovascular outcomes compared with those with type A AAD. These findings suggest that although type A AAD exhibits remarkable severity during the acute phase, appropriate treatment may attenuate this excess risk, resulting in comparable 5-year mortality between subsets of AMI and AAD.

Our study has some limitations. First, mortality data were collected based on death certificates in Shiga Prefecture, Japan, and we were unable to capture information on patients who relocated and died outside this area. This may have led to an underestimation of the true long-term mortality. Second, differences in outcomes between patients who underwent surgical or interventional treatment and those who did not should not be interpreted as evidence for or against the efficacy of the treatment, because patients who were not treated were likely in a more critical condition and therefore unsuitable for such procedures. Therefore, caution is warranted when interpreting prognostic data related to these treatments. Third, because this study was conducted within a single prefecture in Japan, the generalizability of our findings to other regions or patient populations may be limited. Future prospective multicentre studies with detailed treatment data are needed to validate our findings. Fourth, the definition of AMI in this study included type 3 MI, which likely contributed to the relatively high mortality observed in the AMI group. However, because type 3 MI is defined by sudden cardiac death, complete case ascertainment within a registry is inherently challenging. Therefore, the observed mortality in the AMI cohort may be underestimated, and the true mortality could be even higher. Despite these limitations, this study represents the first analysis to directly compare long-term mortality between AMI and AAD within a population-based cohort. By characterizing both the acute and long-term clinical courses of these conditions, our findings provide clinically relevant context for shared decision-making, helping patients and families understand not only the immediate lethality but also the long-term clinical course of AMI and AAD.

## Conclusions

In this population-based study in Japan, patients with AAD experienced substantially higher 5-year mortality than those with AMI, largely driven by excess deaths during the acute phase. Among patients who survived the first 30 days, long-term cardiovascular mortality was comparable between diseases, suggesting that early survival critically determines long-term prognosis. These findings highlight the importance of timely access to definitive surgical or interventional treatment and provide real-world context for understanding prognosis after major acute cardiovascular emergencies.

## Supplementary Material

oeag094_Supplementary_Data

## Data Availability

The data underlying this article cannot be shared publicly due to privacy and ethical restrictions. Data from the Shiga Stroke and Heart Attack Registry may be made available from the corresponding author upon reasonable request and with permission of the registry governance committee.

## References

[1] Reed GW, Rossi JE, Cannon CP. Acute myocardial infarction. Lancet 2017;389:197–210. 27502078 10.1016/S0140-6736(16)30677-8

[2] Magnussen C, Ojeda FM, Leong DP, Alegre-Diaz J, Amouyel P, Aviles-Santa L, De Bacquer D, Ballantyne CM, Bernabé-Ortiz A, Bobak M, Brenner H, Carrillo-Larco RM, De Lemos J, Dobson A, Dörr M, Donfrancesco C, Drygas W, Dullaart RP, Engström G, Ferrario MM, Ferrières J, De Gaetano G, Goldbourt U, Gonzalez C, Grassi G, Hodge AM, Hveem K, Iacoviello L, Kamran Ikram M, Irazola V, Jobe M, Jousilahti P, Kaleebu P, Kavousi M, Kee F, Khalili D, Koenig W, Kontsevaya A, Kuulasmaa K, Lackner KJ, Leistner DM, Lind L, Linneberg A, Lorenz T, Lyngbakken MN, Malekzadeh R, Malyutina S, Mathiesen EB, Melander O, Metspalu A, Jaime Miranda J, Moitry M, Mugisha J, Nalini M, Nambi V, Ninomiya T, Oppermann K, D'Orsi E, Pająk A, Palmieri L, Panagiotakos D, Perianayagam A, Peters A, Poustchi H, Prentice AM, Prescott E, Risérus U, Salomaa V, Sans S, Sakata S, Schöttker B, Schutte AE, Sepanlou SG, Sharma SK, Shaw JE, Simons LA, Söderberg S, Tamosiunas A, Thorand B, Tunstall-Pedoe H, Twerenbold R, Vanuzzo D, Veronesi G, Waibel J, Goya Wannamethee S, Watanabe M, Wild PS, Yao Y, Zeng Y, Ziegler A, Blankenberg S. Global effect of modifiable risk factors on cardiovascular disease and mortality. N Engl J Med 2023;389:1273–1285. 37632466 10.1056/NEJMoa2206916 PMC10589462

[3] Zhou Z, Cecchi AC, Prakash SK, Milewicz DM. Risk factors for thoracic aortic dissection. Genes (Basel) 2022;13:1814. 36292699 10.3390/genes13101814 PMC9602170

[4] Stone PH, Libby P, Boden WE. Fundamental pathobiology of coronary atherosclerosis and clinical implications for chronic ischemic heart disease management—the plaque hypothesis. JAMA Cardiol 2023;8:192. 36515941 10.1001/jamacardio.2022.3926 PMC11016334

[5] Murillo H, Molvin L, Chin AS, Fleischmann D. Aortic dissection and other acute aortic syndromes: diagnostic imaging findings from acute to chronic longitudinal progression. RadioGraphics 2021;41:425–446. 33646901 10.1148/rg.2021200138

[6] Obayashi Y, Shiomi H, Morimoto T, Miyake M, Inoko M, Nishikawa R, Kaneda K, Yamamoto K, Takeji Y, Tada T, Nagao K, Uegaito T, Ehara N, Sakai H, Suwa S, Tamura T, Sakamoto H, Inada T, Matsuda M, Sato Y, Furukawa Y, Ando K, Kadota K, Nakagawa Y, Kimura T. The impact of mitral regurgitation on long-term outcomes in acute myocardial infarction undergoing percutaneous coronary intervention. Am J Cardiol 2023;203:384–393. 37517134 10.1016/j.amjcard.2023.07.038

[7] Masuda M, Nakatani D, Hikoso S, Suna S, Usami M, Matsumoto S, Kitamura T, Minamiguchi H, Okuyama Y, Uematsu M, Yamada T, Iwakura K, Hamasaki T, Sakata Y, Sato H, Nanto S, Hori M, Komuro I, Sakata Y. Clinical impact of ventricular tachycardia and/or fibrillation during the acute phase of acute myocardial infarction on in-hospital and 5-year mortality rates in the percutaneous coronary intervention era. Circ J 2016;80:1539–1547. 27238618 10.1253/circj.CJ-16-0183

[8] Kochar A, Chen AY, Sharma PP, Pagidipati NJ, Fonarow GC, Cowper PA, Roe MT, Peterson ED, Wang TY. Long-term mortality of older patients with acute myocardial infarction treated in US clinical practice. J Am Heart Assoc 2018;7:e007230. 29960995 10.1161/JAHA.117.007230 PMC6064921

[9] Thrane PG, Olesen KKW, Thim T, Gyldenkerne C, Hansen MK, Stødkilde-Jørgensen N, Jakobsen L, Bødtker Mortensen M, Dalby Kristensen S, Maeng M. 10-Year mortality after ST-segment elevation myocardial infarction compared to the general population. J Am Coll Cardiol 2024;83:2615–2625. 38897670 10.1016/j.jacc.2024.04.025

[10] Stødkilde-Jørgensen N, Olesen KKW, Gyldenkerne C, Hansen MK, Nørgaard BL, Thim T, Nielsen RR, Maeng M. Mortality after non-ST-segment elevation myocardial infarction: impact of left ventricular function and coronary artery disease. JACC Adv 2025;4:102155. 40961738 10.1016/j.jacadv.2025.102155 PMC12476103

[11] Melvinsdottir IH, Lund SH, Agnarsson BA, Sigvaldason K, Gudbjartsson T, Geirsson A. The incidence and mortality of acute thoracic aortic dissection: results from a whole nation study. Eur J Cardiothorac Surg 2016;50:1111–1117. 27334108 10.1093/ejcts/ezw235

[12] Howard DPJ, Banerjee A, Fairhead JF, Perkins J, Silver LE, Rothwell PM. Population-based study of incidence and outcome of acute aortic dissection and premorbid risk factor control: 10-year results from the Oxford vascular study. Circulation 2013;127:2031–2037. 23599348 10.1161/CIRCULATIONAHA.112.000483 PMC6016737

[13] Tsai TT, Fattori R, Trimarchi S, Isselbacher E, Myrmel T, Evangelista A, Hutchison S, Sechtem U, Cooper JV, Smith DE, Pape L, Froehlich J, Raghupathy A, Januzzi JL, Eagle KA, Nienaber CA. Long-term survival in patients presenting with type B acute aortic dissection. Circulation 2006;114:2226–2231. 17101856 10.1161/CIRCULATIONAHA.106.622340

[14] Tsai TT, Evangelista A, Nienaber CA, Trimarchi S, Sechtem U, Fattori R, Myrmel T, Pape L, Cooper JV, Smith DE, Fang J, Isselbacher E, Eagle KA. Long-term survival in patients presenting with type A acute aortic dissection. Circulation 2006;114:I-350–I-356. 16820599 10.1161/CIRCULATIONAHA.105.000497

[15] Sawayama Y, Takashima N, Harada A, Yano Y, Yamamoto T, Higo Y, Shioyama W, Fujii T, Tanaka-Mizuno S, Kita Y, Miura K, Nozaki K, Suzuki T, Nakagawa Y. Incidence and in-hospital mortality of acute myocardial infarction: a report from a population-based registry in Japan. J Atheroscler Thromb 2023;30:1407–1419. 36596530 10.5551/jat.63888 PMC10564630

[16] Higo Y, Sawayama Y, Takashima N, Harada A, Yano Y, Yamamoto T, Shioyama W, Fujii T, Tanaka-Mizuno S, Kita Y, Miura K, Nozaki K, Suzuki T, Nakagawa Y. Epidemiology of acute aortic dissection in a general population of 1.4 million people in Japan― shiga stroke and heart attack registry ―. Circ J 2023;87:1155–1161. 37211402 10.1253/circj.CJ-22-0758

[17] Thygesen K, Alpert JS, Jaffe AS, Chaitman BR, Bax JJ, Morrow DA, White HD. Fourth universal definition of myocardial infarction. J Am Coll Cardiol 2018;72:2231–2264. 30153967 10.1016/j.jacc.2018.08.1038

[18] Hiratzka LF, Bakris GL, Beckman JA, Bersin RM, Carr VF, Casey DE, Eagle KA, Hermann LK, Isselbacher EM, Kazerooni EA, Kouchoukos NT, Lytle BW, Milewicz DM, Reich DL, Sen S, Shinn JA, Svensson LG, Williams DM. 2010; ACCF/AHA/AATS/ACR/ASA/SCA/SCAI/SIR/STS/SVM guidelines for the diagnosis and management of patients with thoracic aortic disease. Circulation 2010;121:e266–e369. 20233780 10.1161/CIR.0b013e3181d4739e

[19] Garcia-Garcia HM, McFadden EP, Farb A, Mehran R, Stone GW, Spertus J, Onuma Y, Morel M-, Van Es G-A, Zuckerman B, Fearon WF, Taggart D, Kappetein A-P, Krucoff MW, Vranckx P, Windecker S, Cutlip D, Serruys PW. Standardized End point definitions for coronary intervention trials: the academic research consortium-2 consensus document. Circulation 2018;137:2635–2650. 29891620 10.1161/CIRCULATIONAHA.117.029289

