# Supplementary material for: Comparative long-term prognosis of acute myocardial infarction and acute aortic dissection in a population-based registry
Source: Eur Heart J Open. 2026 May 30;6(3):oeag094. doi: 10.1093/ehjopen/oeag094 (PMC13256082; doi:10.1093/ehjopen/oeag094)
Supplement: oeag094_Supplementary_Data [file oeag094_supplementary_data.docx]

**SUPPLEMENTAL MATERIALS** for **Comparative Analysis of Long-Term Prognosis between Patients with Acute Myocardial Infarction and Acute Aortic Dissection: A Report from a Population-Based Registry in Japan**

Authors: Sawayama et al.

**Supplemental Table 1. Baseline Characteristics by Subtypes**

**Supplemental Table 2. Hazard Ratios for 5-Year All-cause and Cardiovascular Mortality According to Interventional Treatment**

**Supplemental Figure 1. Cumulative Incidence of Death from Any Cause and Cardiovascular Diseases in Patients with AMI and AAD by Subtypes**

**Supplemental Figure 2. Landmark Analysis Beyond 30 Days by Subtypes**

**Supplemental Figure 3. Age-specific estimated mortality in AMI and AAD**

**Supplemental Table 1. Baseline Characteristics by Subtypes**

|  | STEMI  (N = 902) | NSTEMI  (N = 464) | SCD due to MI  (N = 184) | Type A AAD  (N = 219) | Type B AAD  (N = 154) |
| --- | --- | --- | --- | --- | --- |
| Age, years | 70.3 ± 13.2 | 70.2 ± 12.4 | 78.7 ± 13.1 | 75.0 ± 12.8 | 69.9 ± 15.0 |
| Men | 637 (70.6%) | 336 (72.4%) | 117 (63.6%) | 83 (37.9%) | 110 (71.4%) |
| Height | 161.3 ± 10.0  (n=838) | 161.7 ± 9.9  (n=450) | 157.9 ± 11.0  (n=29) | 156.6 ± 10.9  (n=148) | 162.1 ± 9.8  (n=135) |
| BMI | 23.5 ± 3.7  (n=832) | 23.9 ± 3.9  (n=440) | 21.0 ± 3.9  (n=24) | 23.1 ± 4.7  (n=143) | 23.5 ± 4.8  (n=131) |
| Current smoking | 311/852 (36.5%) | 137/446 (30.7%) | 6/38 (15.8%) | 25/141 (17.7%) | 54/143 (37.8%) |
| Current drinking | 308/801 (38.5%) | 174/429 (40.6%) | 14/39 (35.9%) | 33/127 (26.0%) | 56/134 (41.8%) |
| Living alone | 115 (12.8%) | 50 (10.8%) | 7 (3.8%) | 22 (10.1%) | 21 (13.6%) |
| **Comorbidities** |  |  |  |  |  |
| Hypertension | 538/896 (60.0%) | 316/462 (68.4%) | 60/128 (46.9%) | 124/202 (61.4%) | 104/152 (68.4%) |
| Diabetes | 358/900 (39.8%) | 206/462 (44.6%) | 49/131 (37.4%) | 48/200 (24.0%) | 25/152 (16.5%) |
| Dyslipidemia | 578/899 (64.3%) | 319/463 (71.1%) | 32/131 (24.4%) | 65/202 (32.2%) | 70/152 (46.1%) |
| Prior MI | 75/895 (8.4%) | 49/460 (10.7%) | 6/129 (4.7%) | 5/199 (2.5%) | 3/152 (2.0%) |
| Prior AoD | 17/893 (1.9%) | 14/461 (3.0%) | 1/127 (0.8%) | 19/198 (9.6%) | 22/149 (14.8%) |
| Prior ischemic stroke | 77/894 (8.6%) | 48/461 (10.4%) | 14/131 (10.7%) | 19/200 (9.5%) | 9/152 (5.9%) |
| Prior intracerebral bleeding | 19/895 (2.1%) | 4/459 (0.9%) | 3/128 (2.3%) | 8/199 (4.0%) | 5/152 (3.3%) |
| Prior PCI | 108/892 (12.1%) | 89/457 (19.5%) | 9/129 (7.0%) | 6/199 (3.0%) | 8/152 (5.3%) |
| Prior CABG | 5/888 (0.6%) | 8/456 (1.8%) | 4/129 (3.1%) | 0/199 (0%) | 0/152 (0%) |
| **Blood pressure and heart rate on hospital arrival** |  |  |  |  |  |
| SBP, mmHg | 137.9 ± 33.1  (n=855) | 144.6 ± 32.7  (n=448) | 109.8 ± 49.4  (n=16) | 129.0 ± 36.0  (n=138) | 166.0 ± 33.9  (n=146) |
| DBP, mmHg | 82.2 ± 22.0  (n=851) | 82.8 ± 20.5  (n=447) | 62.5 ± 29.6  (n=14) | 69.8 ± 22.6  (n= 138) | 93.5 ± 21.1  (n= 146) |
| Pulse rate, /min | 78.8 ± 22.8  (n=853) | 81.0 ± 19.7  (n=449) | 76.8 ± 34.1  (n=20) | 77.7 ± 20.7  (n=135) | 80.2 ± 18.3  (n=146) |
| **States at hospital arrival** |  |  |  |  |  |
| CPA | 81/902 (9.0%) | 23/464 (5.0%) | 146/153 (95.4%) | 92/219 (42.0%) | 11/154 (7.1%) |
| Japan coma scale |  |  |  |  |  |
| Ⅰ | 764/883 (86.5%) | 432/459 (94.1%) | 8/135 (5.9%) | 115/188 (61.2%) | 135/153 (88.2%) |
| Ⅱ | 52/883 (5.9%) | 10/459 (2.2%) | 1/135 (0.7%) | 15/188 (8.0%) | 9/153 (5.9%) |
| Ⅲ | 67/883 (7.6%) | 17/459 (3.7%) | 126/135 (93.3%) | 58/188 (30.9%) | 9/153 (5.9%) |
| **Treatment for AMI** |  |  |  |  |  |
| No interventional therapy | 78 (8.7%) | 30 (6.5%) | 184 (100%) |  |  |
| Percutaneous coronary intervention | 792 (87.8%) | 387 (83.4%) | 0 (0%) |  |  |
| Coronary artery bypass grafting | 32 (3.6%) | 47 (10.1%) | 0 (0%) |  |  |
| **Treatment for AAD** |  |  |  |  |  |
| No interventional therapy |  |  |  | 120 (54.8%) | 133 (86.4%) |
| Endovascular therapy |  |  |  | 0 (0%) | 6 (3.9%) |
| Surgical therapy |  |  |  | 99 (45.2%) | 15 (9.7%) |

AoD, aortic diseases; AAD, acute aortic dissection; CABG, coronary artery bypass graft; CPA, cardiopulmonary arrest; DBP, diastolic blood pressure; eGFR, estimated glomerular filtration rate; ER, emergency room; MI, myocardial infarction. NSTEMI, non-ST-elevation myocardial infarction; PCI, percutaneous coronary intervention; SBP, systolic blood pressure; SCD, sudden cardiac death; STEMI, ST-elevation myocardial infarction.

**Supplemental Table 2. Hazard Ratios for 5-Year All-cause and Cardiovascular Mortality According to Interventional Treatment**

|  | All-cause mortality | | Cardiovascular mortality | |
| --- | --- | --- | --- | --- |
|  | Adjusted HR  (95% CI) | P value | Adjusted HR  (95% CI) | P value |
| STEMI | 3.55 (2.56–4.91) | <0.001 | 4.39 (3.03–6.35) | <0.001 |
| NSTEMI | 3.38 (1.96–5.82) | <0.001 | 3.60 (1.82–7.13) | <0.001 |
| Type A AAD | 6.24 (3.90–10.0) | <0.001 | 8.36 (4.80–14.6) | <0.001 |
| Type B AAD | 0.65 (0.31–1.39) | 0.264 | 0.49 (0.20–1.21) | 0.121 |

Hazard ratios (HRs) and 95% confidence intervals (CIs) were calculated with interventional treatment group as the reference group using a multivariable Cox proportional hazards model adjusting for age and sex.

AAD, acute aortic dissection; NSTEMI, non-ST-elevation myocardial infarction; STEMI, ST-elevation myocardial infarction.

**Supplemental Figure 1. Cumulative Incidence of Death from Any Cause and Cardiovascular Diseases in Patients with AMI and AAD by Subtypes**

**
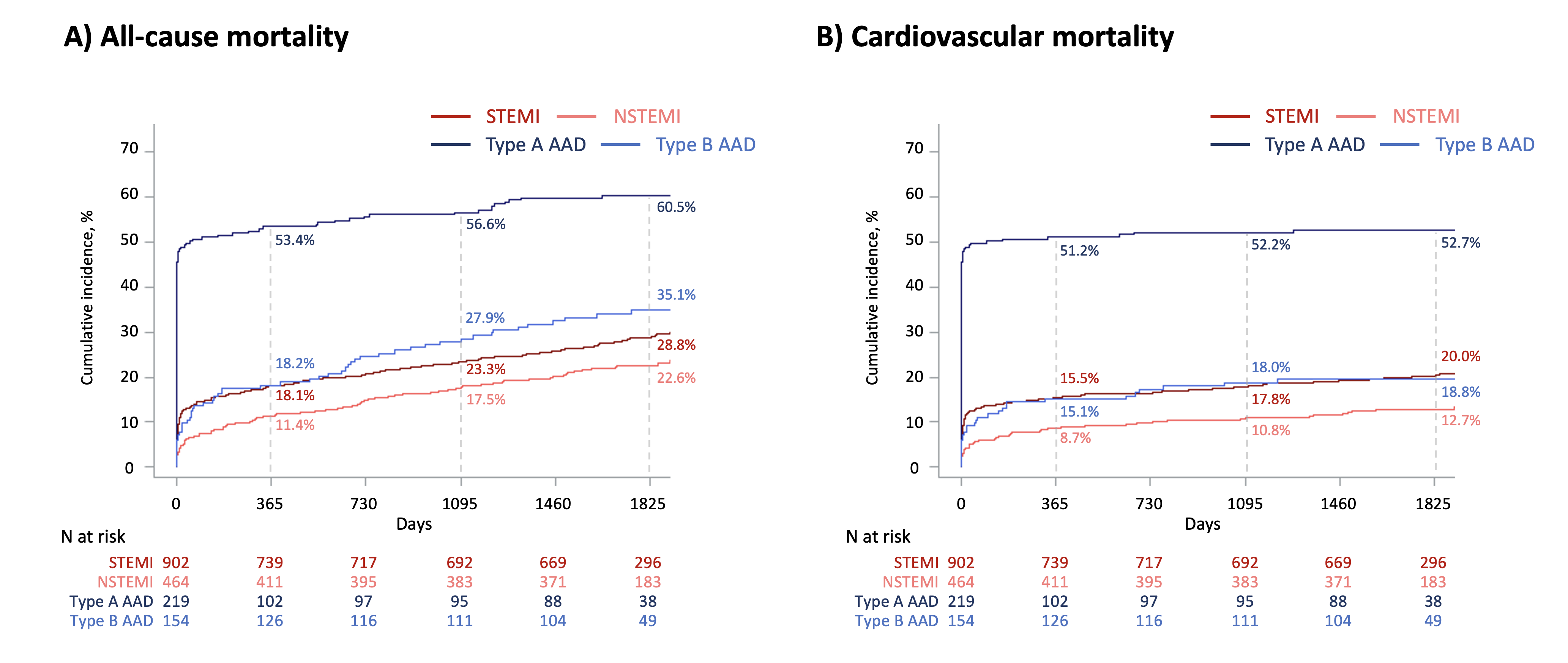
**

Kaplan–Meier curves between patients with STEMI (dark red), NSTEMI (light red), type A AAD (dark blue), and type B AAD (light blue). Cumulative incidence of death from (A) any cause and (B) cardiovascular diseases. Dashed vertical lines indicate the cumulative event rates at 1-, 3-, and 5-year time points.

AAD, acute aortic dissection; NSTEMI, non-ST-elevation myocardial infarction; STEMI, ST-elevation myocardial infarction.

**Supplemental Figure 2. Landmark Analysis Beyond 30 Days s by Subtypes**

**
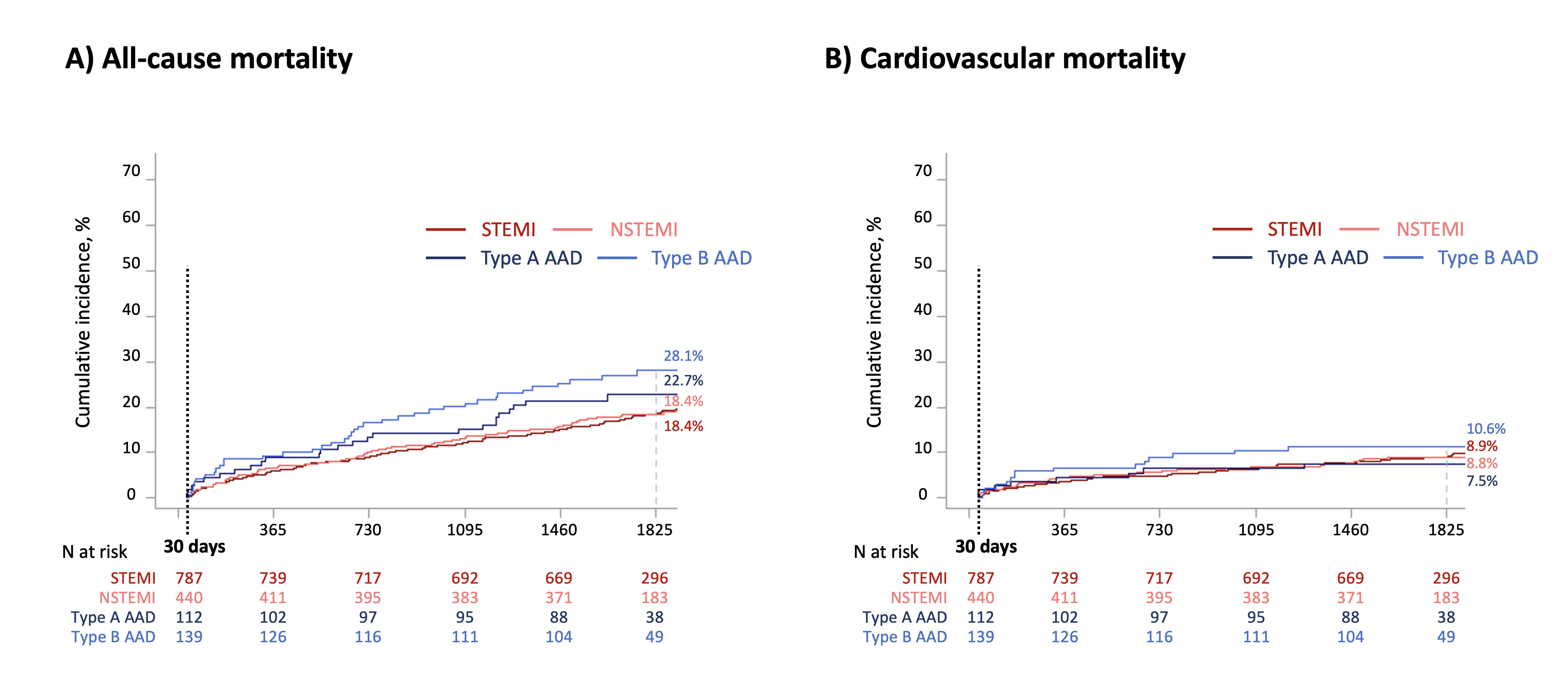
**

Patients who died within 30 days after the onset were excluded from the landmark analysis beyond 30 days. The color coding is as follows: STEMI (dark red), NSTEMI (light red), type A AAD (dark blue), and type B AAD (light blue).

AAD, acute aortic dissection; NSTEMI, non-ST-elevation myocardial infarction; STEMI, ST-elevation myocardial infarction.

**
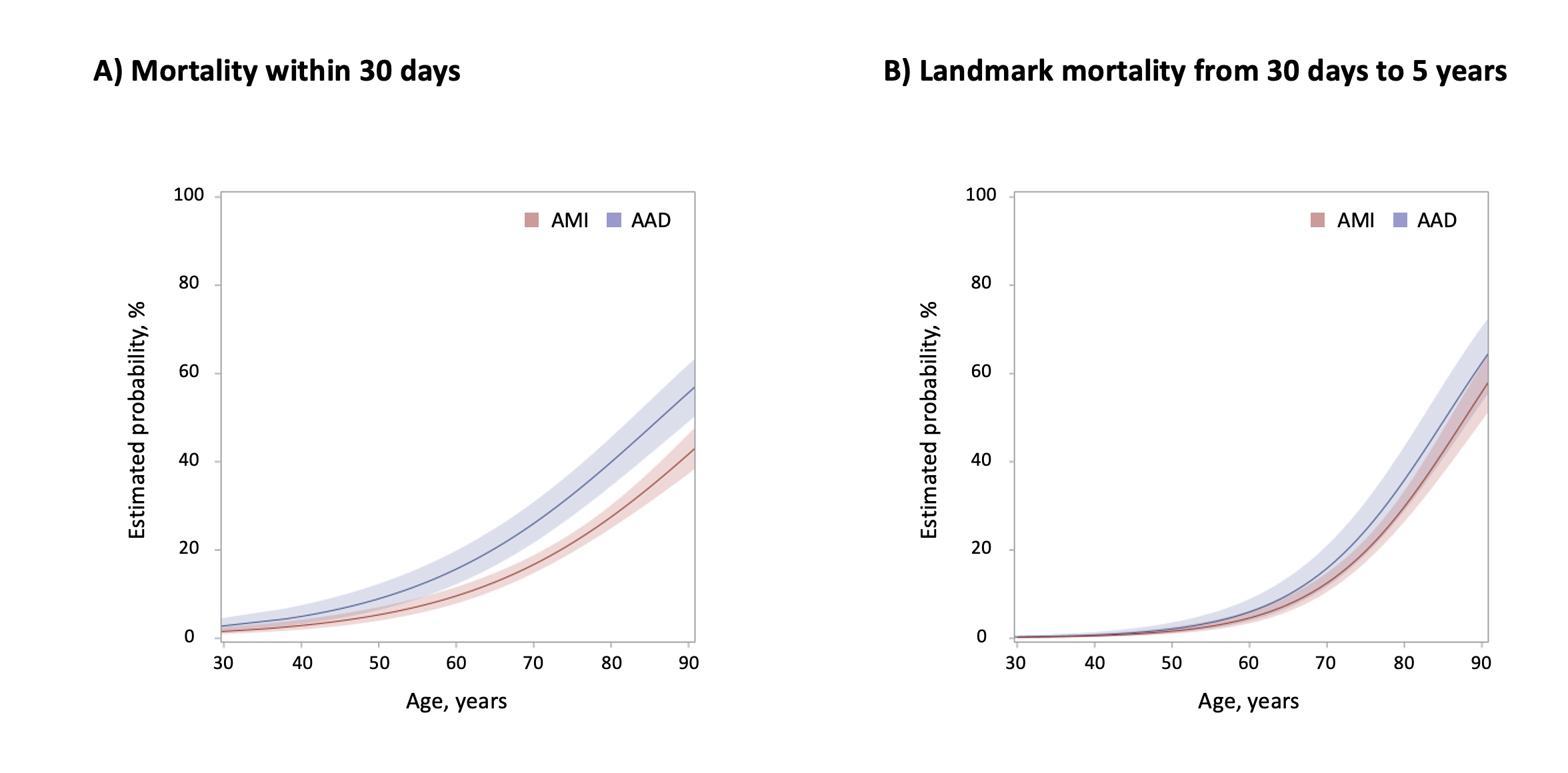
Supplemental Figure 3. Age-specific estimated mortality in AMI and AAD.**

Estimated probability of (A) mortality within 30 days and (B) landmark mortality from 30 days to 5 years between patients with AMI (red) and AAD (blue). Shaded regions indicate 95% confidence intervals. Probabilities were estimated using logistic regression models.

AAD, acute aortic dissection; AMI, acute myocardial infarction.
